# New Insight into Visible-Light-Driven Photocatalytic Activity of Ag-Loaded and Oxygen Vacancy-Containing BiOBr_(OV)_/BiOI_0.08_ Microspheres

**DOI:** 10.3390/ma17246297

**Published:** 2024-12-23

**Authors:** Xiaobin Hu, Mingxing Zhao, Rongfei Zhang

**Affiliations:** School of Life Science, Huzhou University, Huzhou 313000, China

**Keywords:** Ag/BiOBr_(OV)_/BiOI_0.08_, silver loading, oxygen vacancy, carrier migration, photocatalysis

## Abstract

A series of Ag-loaded and oxygen vacancy (OV)-containing BiOBr_(OV)_/BiOI_0.08_ (Ag/BiOBr_(OV)_/BiOI_0.08_) photocatalysts with varying Ag loading levels were synthesized via the solvothermal–photocatalytic reduction method. As confirmed via optical, photoelectrochemical, and 4-chlorophenol photodegradation experiments, a low Ag loading level significantly enhanced the photogenerated charge carrier (PCC) transfer on the BiOBr_(OV)_/BiOI_0.08_ semiconductor surface and the performance of Ag/BiOBr_(OV)_/BiOI_0.08_ photocatalysts, which was attributable to the synergism between the effect of OVs and the localized surface plasmon resonance (LSPR) of Ag nanoparticles. Additionally, BiOBr_(OV)_/BiOI heterojunctions facilitated efficient visible-light harvesting and PCC separation. As indicated by finite-difference time-domain (FDTD) simulations and density functional theory (DFT) calculations, the electric field intensity in the “hot spots” generated at the interface between the BiOBr_(OV)_/BiOI_0.08_ semiconductor and Ag nanoparticles increased by more than eight times, and the presence of OVs and Ag atomic clusters introduced impurity energy levels in the semiconductor bandgap, improving PCC separation and Ag/BiOBr_(OV)_/BiOI_0.08_ photocatalytic efficiency. However, an increase in silver loading renders the composite metallic, suggesting a reduction in its photocatalytic performance. This work provides new insights for designing highly active visible light catalysts and contributes to the development of more efficient plasmonic catalysts.

## 1. Introduction

Photocatalysis is a highly promising green technology for which its potential applications in organic pollutant degradation and solar energy conversion have garnered significant attention [1,2,3,4,5,6]. However, its implementation is hindered by the limited photon absorption and photogenerated charge carrier (PCC) separation/migration efficiencies of existing photocatalysts. Consequently, developing photocatalysts capable of efficiently capturing photon energy and facilitating PCC separation and migration is a pivotal issue in photocatalysis research [7,8].

Single-component photocatalysts generally exhibit high PCC recombination and low visible-light utilization rates; thus, various methods have been employed to augment their photocatalytic performance, including morphology modulation [2,9,10], noble metal deposition [11,12,13], ion doping [14,15,16], solid solution formation [17,18], heterostructure construction [5,19,20,21], and oxygen defect introduction [9,18,22,23]. Noble metal deposition is an extensively used photocatalytic-performance-augmenting method [24,25,26]. On one hand, it is generally believed that noble metal nanoparticles in metal/semiconductor heterostructures act as reservoirs of photogenerated electrons, thereby promoting an interfacial charge-transfer process [27,28]. After their photoexcitation from the valence band (VB) to the conduction band (CB) of the semiconductor, these electrons are readily transferred to the noble metal, thus improving electron–hole separation. On the other hand, photogenerated holes in the semiconductor VB can oxidize water, degrade organic pollutants, or produce reactive oxygen radicals. Utilizing the localized surface plasmon resonance (LSPR) of metal nanostructures is another widely employed method for enhancing photocatalyst performance [29,30,31,32]. LSPR is an optical property unique to metal nanoparticles. It represents strong photon absorption or dispersion occurring when the incident light frequency corresponds to the cumulative oscillation frequency of free electrons on the metal surface. The free electrons absorb photons with the same frequency as the resonance frequency, gain energy, overcome the Schottky barrier at the metal/semiconductor interface, and are then excited to the semiconductor CB. Furthermore, after photon absorption, the plasma resonance intensity is significantly amplified, resulting in an extremely strong electric field intensity within a radius of tens of nanometers around the metal nanoparticles, which is sufficient to enable the semiconductor within this influenced range to achieve the spatial separation of positive and negative charges [33]. Therefore, the mechanism by which metal nanoparticles in metal/semiconductor heterostructures influence the performance of metal-loaded semiconductor-based photocatalysts requires further investigation [30,31,32].

Bismuth oxyhalides (BiOX, X = Cl, Br, and I), which are ternary metal oxide semiconductors, possess a layered structure composed of positively charged [Bi_2_O_2_]^2+^ sublayers sandwiched between two negatively charged X^−^ sublayers [34,35]. This structure can create an internal static electric field between the positively and negatively charged sublayers, inducing PCC separation and enhancing BiOX photocatalytic performance [36]. Thus, these semiconductors have received considerable attention. Heterojunction formation is conducive to the PCC transfer between two semiconductor interfaces, thus improving PCC separation efficiency and photocatalyst performance. BiOX-based heterojunctions have been synthesized and studied [19,37,38,39], and researchers have shown that BiOBr/BiOI heterojunctions exhibit enhanced PCC separation and organic contaminant photocatalytic degradation efficiencies [40,41]. Moreover, researchers have demonstrated that oxygen vacancies (OVs) on the BiOX (001) surface can alter local electronic states and act as charge traps, thereby preventing PCC recombination and enhancing BiOX photocatalytic activity [18,42]. Ag has been commonly employed as a cocatalyst for semiconductor-based photocatalysts because it can augment visible-light-driven photocatalysis through its LSPR and promote PCC separation [43,44,45].

In this study, we prepared a novel plasmonic catalyst comprising Ag nanoparticles deposited on an OV-containing BiOBr_(OV)_/BiOI_0.08_ composite. The Ag/BiOBr_(OV)_/BiOI_0.08_ photocatalysts were comprehensively characterized and then employed for degrading 4-chlorophenol (4-CP) under visible-light irradiation. In order to reveal the underlying reasons for the significant impact of changes in Ag loading on the photocatalytic performance of the catalyst, the role of Ag loading was further investigated via finite-difference time-domain (FDTD) simulations and density functional theory (DFT) calculations. The surface electronic structures of OV containing BiOBr_(OV)_/BiOI_0.08_ composites loaded with Ag clusters and Ag atomic layers were compared. It was revealed that when the loading amount of Ag was very low, the combination of Ag with OV induced impurity energy levels on the surface of the composite catalyst, which facilitated the separation of photogenerated charge carriers. However, increasing the loading of Ag, together with OV, results in the formation of excessive and significant impurity energy levels on the surface of the composite catalyst, which overlaps with the valence band and conduction band. This may act as recombination centers for photogenerated charges, thus hindering photocatalysis.

## 2. Materials and Methods

### 2.1. Materials

Deionized water (H_2_O) and analytical-grade reagents (without additional purification) were used in all experiments. Absolute ethanol (CH_3_CH_2_OH), bismuth nitrate pentahydrate (Bi(NO_3_)_3_·5H_2_O), nitric acid (HNO_3_), potassium iodide (KI), silver nitrate (AgNO_3_), and polyvinylpyrrolidone (PVP) were obtained from Sinopharm Chemical Reagent Co., Ltd. (Shanghai, China). Glycerol (CH_2_OHCHOHCH_2_OH), acetic acid (CH_3_COOH), and 4-CP were acquired from Shanghai Zhanyun Chemical Co., Ltd. (Shanghai, China).

### 2.2. Photocatalyst Preparation

#### 2.2.1. Synthesis of BiOI, BiOBr_(OV)_, and BiOBr_(OV)_/BiOI_0.08_ Photocatalysts

Synthesis of BiOI nanoparticles: In a beaker, Bi(NO_3_)_3_·5H_2_O (1.94 g) was dissolved in CH_3_COOH (10 mL) using ultrasonic agitation. Concurrently, in another beaker, KI (0.66 g) was dissolved in H_2_O (100 mL). The Bi(NO_3_)_3_ solution was gradually added to the KI solution under rapid stirring, which was continued for another 3–4 h. Following filtration, the resulting BiOI product was washed thrice each with H_2_O and CH_3_CH_2_OH and subsequently dried at 80 °C for 12 h.

Synthesis of BiOBr_(OV)_/BiOI_0.08_ nanoparticles: Glycerol (20 mL) was added to H_2_O (20 mL). Then, Bi(NO_3_)_3_·5H_2_O (0.972 g) and PVP (0.800 g) were added to the glycerol solution, and the mixture was stirred for 1 h to obtain solution A. Separately, 0.206 g of NaBr and 0.056 g of previously synthesized BiOI nanoparticles were dispersed into another 10 mL of H_2_O under ultrasonic conditions to obtain suspension B. Over a period of 1 h, suspension B was gradually introduced into solution A under continuous stirring at ambient temperatures, resulting in suspension C. Following the 150 °C 8 h hydrothermal treatment (in a 50 mL Teflon-lined stainless-steel autoclave), natural cooling to room temperature, and filtration of suspension C, the BiOBr_(OV)_/BiOI_0.08_ product was washed thrice each with H_2_O and CH_3_CH_2_OH and subsequently dried at 80 °C for 12 h.

BiOBr_(OV)_ was synthesized similarly to BiOBr_(OV)_/BiOI_0.08_, except that BiOI was omitted from suspension B.

#### 2.2.2. Synthesis of Ag/BiOBr_(OV)_/BiOI_0.08_ Photocatalysts

Ag/BiOBr_(OV)_/BiOI_0.08_ nanoparticles with different Ag loading levels (0.2% Ag/BiOBr_(OV)_/BiOI_0.08_, 0.5% Ag/BiOBr_(OV)_/BiOI_0.08_, 1.0% Ag/BiOBr_(OV)_/BiOI_0.08_, and 1.5% Ag/BiOBr_(OV)_/BiOI_0.08_ nanoparticles) were synthesized by depositing Ag nanoparticles on BiOBr_(OV)_/BiOI_0.08_ nanoparticles via a photoreduction method. For the preparation of 1.0% Ag/BiOBr_(OV)_/BiOI_0.08_ nanoparticles, for which the Ag to BiOBr_(OV)_/BiOI_0.08_ molar ratio was 1:100, AgNO_3_ (0.0055 g) was dissolved in methanol (50 mL) under continuous stirring, and BiOBr_(OV)_/BiOI_0.08_ nanoparticles (1 g) were added to the methanol solution. The resulting suspension underwent ultrasonic treatment for 2 min followed by ultraviolet lamp (λ = 365 nm, 30 W) irradiation for 6 min under vigorous stirring [44]. The formation of Ag/BiOBr_(OV)_/BiOI_0.08_ nanoparticles was enabled via Ag^+^ photocatalytic reduction. Following filtration, the product was rinsed thrice each with H_2_O and CH_3_CH_2_OH and subsequently dried at 80 °C for 2 h.

### 2.3. Photocatalyst Characterization

Photocatalyst morphological characteristics were determined using a Talos F200X transmission electron microscope (Thermo Scientific, Waltham, MA, USA), photocatalyst microstructural imaging was performed using a SUPRA 40 scanning electron microscope (Carl Zeiss Microscopy GmbH, Munich, Germany), and elemental mapping was conducted via energy-dispersive X-ray spectroscopy (EDS) using an XFlash 6–10 detector (Bruker, Heidelberg, Germany). The X-ray diffraction (XRD) patterns of the synthesized photocatalysts were acquired with a D8 Advance X-ray diffractometer (Bruker, Germany; Cu Kα radiation (λ = 1.5418 Å) and a 5°/minute scan rate), and the surface elemental composition and valence states of the photocatalysts were examined with an ESCALAB 250Xi X-ray photoelectron spectrometer (XPS; Thermo Fisher Scientific, USA; monochromatic Al Kα X-ray radiation (1486.71 eV)). The specific surface area of each photocatalyst was measured at 77 K using an ASAP 2020 analyzer (Micromeritics Instrument Corporation, Norcross, GA, USA), and reactive oxygen species (ROS) generation was assessed using an EMX 10/12 electron spin resonance (ESR) spectrometer (Bruker, Germany). Hydroxyl (‧OH) and superoxide anion (‧O_2_^−^) radicals were trapped using 5,5-dimethyl-1-pyrroline-N-oxide (DMPO), and singlet oxygen (^1^O_2_) was trapped using 2,2,6,6-tetramethylpiperidine (TEMP). UV-vis DRS spectra were recorded using a UV270 spectrophotometer (Shimadzu, Kyoto, Japan), with BaSO_4_ being the reflectance standard, while steady-state photoluminescence (PL) spectra were obtained using an FLS1000 photoluminescence spectrophotometer (Edinburgh Instruments, Livingston, UK).

### 2.4. Photoelectrochemical Measurements

The surface photocurrent of each photocatalyst was measured with a three-electrode configuration using a CHI660E electrochemical workstation (Shanghai Chenhua, Shanghai, China), with a 0.5 M aqueous Na_2_SO_4_ solution and a 500 W xenon lamp with a λ ≥ 400 nm cutoff filter being the electrolyte and illumination source, respectively. Photocatalyst powder (10 mg) was added to anhydrous methanol (1 mL), the dispersion was sonicated for 30 min, the resulting suspension (150 μL) was applied to an ITO glass substrate, and the coated substrate was dried at ambient temperature. The photocatalyst-coated ITO glass, a platinum foil, and a calomel electrode functioned as the working, counter, and reference electrodes, respectively.

Transient surface photovoltage (TPV) spectra were acquired using a CEL-TPV2000 electrochemical workstation (Beijing China Education AuLight Co., Ltd., Beijing, China). The light source emitted a pulsed laser with a 355 nm wavelength, 5 ns pulse width, and 30 μJ/pulse intensity.

### 2.5. Photocatalytic Degradation Experiments

A photocatalyst (20 mg) was precisely weighed and added to a 10 mg/L aqueous 4-CP solution (100 mL), and the dispersion was sonicated for 2 min. Following stirring in the dark for 60 min (to realize adsorption–desorption equilibrium on the photocatalyst surface), 4-CP photocatalytic degradation was commenced by irradiating the suspension with a 300 W xenon lamp equipped with a λ ≥ 400 nm cutoff filter. The distance of the liquid surface from the irradiation source was maintained at 15 cm, and the temperature of the suspension was maintained with a cold trap utilizing constant-temperature cooling water.

Aliquots were sampled from the reaction suspension at certain intervals and passed through a 0.22 μm membrane filter prior to their use for HPLC analysis. An LC-16 instrument (Shimadzu, Japan) and a methanol–water (70:30, *v*/*v*) mobile phase were employed for the HPLC analysis, which was conducted at 282 nm with a 1 mL/minute flow rate.

### 2.6. FDTD Simulations

Lumerical FDTD Solutions 7.5 was used to execute three-dimensional full-field FDTD simulations by introducing an electromagnetic pulse spanning the 400 nm to 1000 nm wavelength range into a computational domain encompassing the target nanostructure, which was enclosed by an appropriately sized virtual boundary. A 0.5 nm mesh size was selected to obtain the extinction spectra and calculate the electric field (EF) intensity enhancements for Ag/BiOBr_(OV)_/BiOI_0.08_ photocatalysts. The refractive index of BiOBr/BiOI_0.08_ was specified at 2.19 according to the study by Dai and Zhao [46], while the dielectric function of Ag was adopted from a paper by Johnson and Christy [47]. After field vector monitoring at three-dimensional grid points, the absorption spectra of BiOBr/BiOI and Ag were extracted, and the field distribution maps for specific wavelengths were generated.

### 2.7. Periodic DFT Calculations

The GGA-PBE functional was applied in the Vienna Ab initio Simulation Package 6.1.2 as the exchange-correlation functional for the computation of geometry, band structure, and density of states (DOSs). A 400 eV plane-wave cutoff energy was designated for geometry optimization and electronic structure calculations. The total energy and residual force convergence criteria were specified to 10^−4^ eV and 10^−2^ eV/Å, respectively, and Brillouin zones were sampled using a 3 × 3 × 1 Monkhorst-Pack mesh.

A slab model for BiOBr and BiOI crystals oriented in the (001) direction was constructed. The BiOBr/BiOI and BiOBr_(OV)_/BiOI structures utilized for DFT calculations are depicted in Figures S1 and S2, respectively. For Ag/BiOBr_(OV)_/BiOI_0.08_ photocatalysts, the BiOBr_(OV)_ surface with an overlaying Ag atomic cluster or layer was selected as the model for DOS and band structure calculations. The Ag(cluster)/BiOBr_(OV)_ and Ag(layer)/BiOBr_(OV)_ structures used for DFT calculations are illustrated in Figures S3 and S4, respectively.

## 3. Results and Discussion

### 3.1. Characterization Results

**Figure 1:**
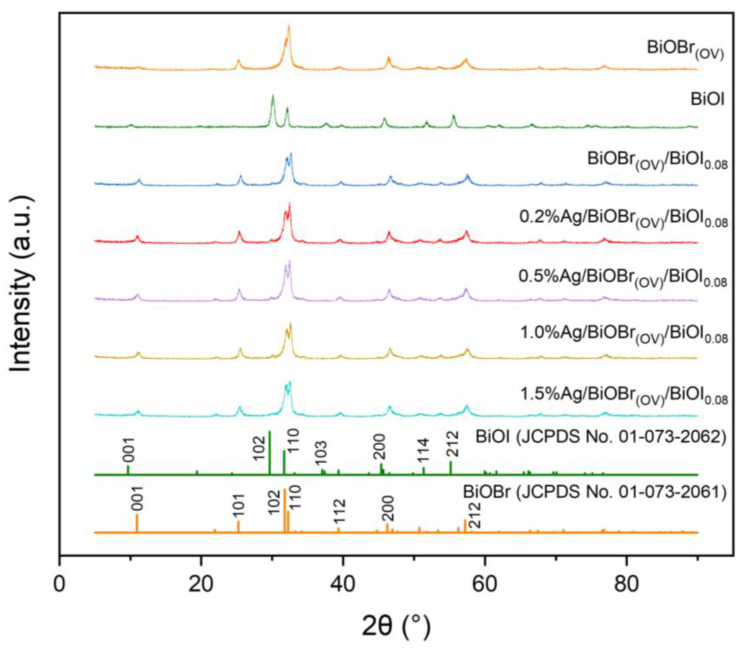
XRD patterns of BiOBr_(OV)_, BiOI, 0.2% Ag/BiOBr_(OV)_/BiOI_0.08_, 0.5% Ag/BiOBr_(OV)_/BiOI_0.08_, 1.0% Ag/BiOBr_(OV)_/BiOI_0.08_, and 1.5% Ag/BiOBr_(OV)_/BiOI_0.08_.

The XRD pattern of BiOBr_(OV)_ (Figure 1) was in alignment with that of tetragonal BiOBr (JCPDS no. 01-073-2061) with the P4/NMM (129) space group [48], and its 2*θ* = 11.34°, 25.32°, 31.86°, 32.36°, 39.46°, 46.48°, and 57.34° diffraction peaks corresponded to the {001}, {101}, {102}, {110}, {112}, {200}, and {212} crystal planes of BiOBr_(OV)_, respectively. The unit cell parameters for the synthesized BiOBr_(OV)_ were a = b = 3.923 Å and c = 8.092 Å.

The XRD pattern of BiOI was consistent with that of tetragonal BiOI (JCPDS no. 01-073-2062) [18,48], and its 2*θ* = 10.16°, 30.10°, 32.14°, 37.62°, 45.86°, 51.78°, and 55.56° diffraction peaks corresponded to the {001}, {102}, {110}, {103}, {200}, {114}, and {212} crystal planes of BiOI, respectively. The unit cell parameters for the synthesized BiOI were a = b = 3.984 Å and c = 9.128 Å.

The BiOI to BiOBr_(OV)_ ratio of BiOBr_(OV)_/BiOI_0.08_ was 0.08:1. Consequently, in the XRD spectrum of BiOBr_(OV)_/BiOI_0.08_, the intensity of each diffraction peak of BiOI is markedly weaker compared to that of BiOBr_(OV)_, with only the most pronounced diffraction peaks being discernible. The XRD spectrum contains the diffraction peaks of both BiOBr_(OV)_ and BiOI without any additional impurity peaks. These BiOBr_(OV)_ and BiOI diffraction peaks were sharp and intense, indicating that BiOBr_(OV)_/BiOI_0.08_ exhibited good crystallinity.

Compared to the XRD pattern of BiOBr_(OV)_/BiOI_0.08_, those of Ag/BiOBr_(OV)_/BiOI_0.08_ photocatalysts exhibited no significant change in BiOBr_(OV)_ and BiOI characteristic diffraction peaks. Almost no Ag diffraction peak was observed in Ag/BiOBr_(OV)_/BiOI_0.08_ XRD patterns, which was attributed to the low concentration and high dispersion of the Ag nanoparticles.

**Figure 2:**
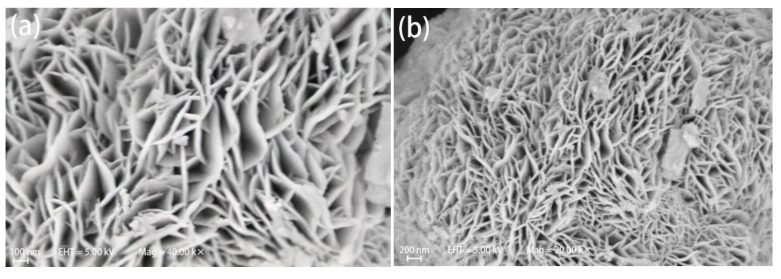

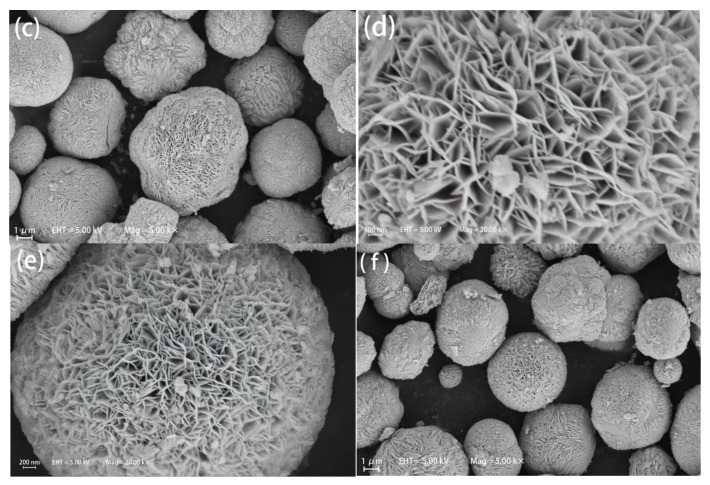
SEM images of BiOBr_(OV)_/BiOI_0.08_ (**a**–**c**) and 0.5% Ag/BiOBr_(OV)_/BiOI_0.08_ (**d**–**f**) photocatalysts.

The flower-like microspheres of BiOBr_(OV)_/BiOI_0.08_ are illustrated in Figure 2a–c. These microspheres were composed of two-dimensional nanosheets that were 0.5 μm to 2.0 μm wide and 10 nm to 20 nm thick (Figure 2a,b), and their average diameter was 3–6 μm. Figure 2d–f depict the morphology and microstructure of the 0.5% Ag/BiOBr_(OV)_/BiOI_0.08_ sample. Morphologically, the 0.5% Ag/BiOBr_(OV)_/BiOI_0.08_ sample does not significantly differ from BiOBr_(OV)_/BiOI_0.08_, also presenting a flower-ball microstructure.

**Figure 3:**
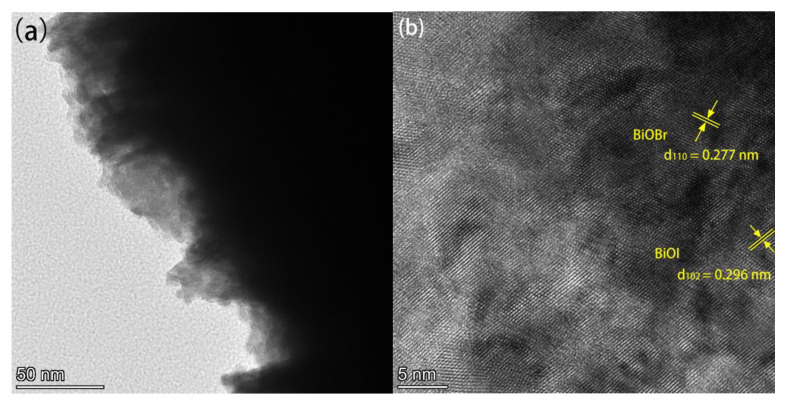

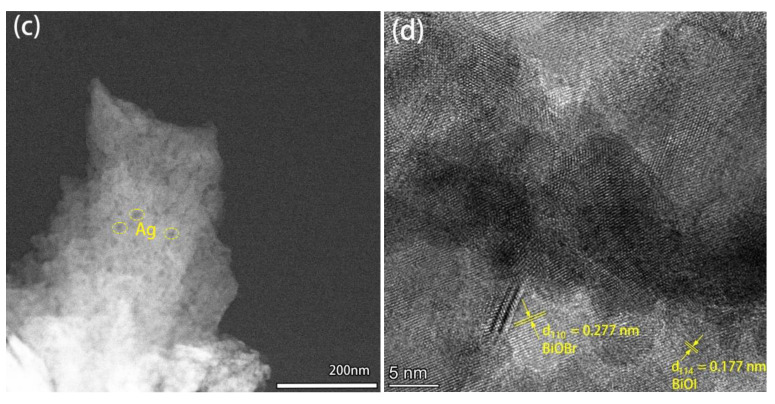
TEM and HRTEM images of BiOBr_(OV)_/BiOI_0.08_ (**a**,**b**) and 0.5% Ag/BiOBr_(OV)_/BiOI_0.08_ (**c**,**d**) photocatalysts.

Figure 3a,b present the TEM and HRTEM images of BiOBr_(OV)_/BiOI_0.08_, which were obtained from the edge of a BiOBr_(OV)_/BiOI_0.08_ nanosheet. The photocatalyst exhibited high crystallinity and distinct lattice fringes. Its continuous lattice fringes with a 0.277 nm interplanar spacing corresponded to the {110} plane of tetragonal BiOBr, while its 0.296 nm and 0.177 nm lattice spacings corresponded to the {102} and {114} planes of tetragonal BiOI, respectively. Figure 3c,d present the TEM and HRTEM images of 0.5% Ag/BiOBr_(OV)_/BiOI_0.08_. The black spots with a 10 nm to 20 nm average diameter in the TEM image (Figure 3c) were likely to be Ag nanoparticles deposited on BiOBr_(OV)_/BiOI_0.08_ nanosheets.

**Figure 4:**
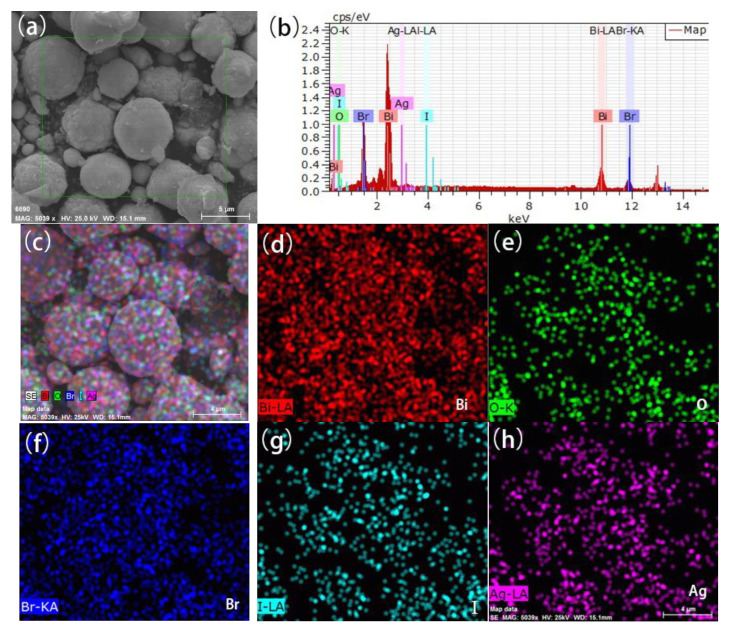
The SEM image (**a**), EDS spectrum (**b**), and elemental maps (overlay (**c**), Bi (**d**), O (**e**), Br (**f**), I (**g**), and Ag (**h**)) of 0.5% Ag/BiOBr_(OV)_/BiOI_0.08_.

The elemental analysis of the 0.5% Ag/BiOBr_(OV)_/BiOI_0.08_ sample is depicted in Figure 4. The photocatalyst contained Bi, O, Br, I, and Ag elements, confirming its purity. Furthermore, the distribution of these elements is highly uniform, suggesting an even dispersion of silver nanoparticles across the BiOBr_(OV)_/BiOI_0.08_ composite microspheres.

**Figure 5:**
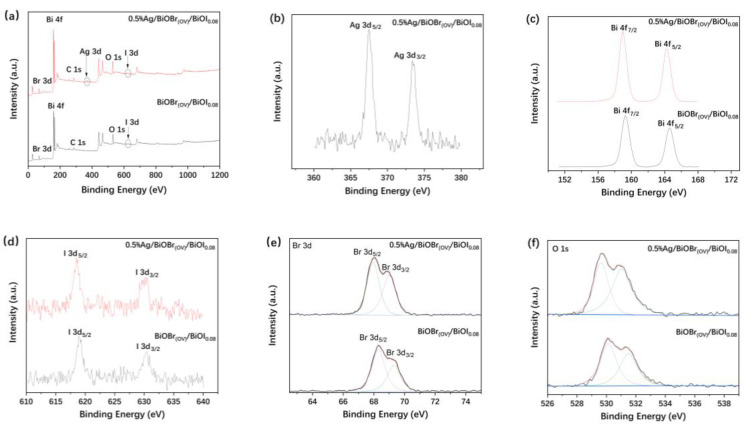
Survey (**a**), Ag 3d (**b**), Bi 4f (**c**), I 3d (**d**), Br 3d (**e**), and O 1s (**f**) XPS spectra of BiOBr_(OV)_/BiOI_0.08_ and 0.5% Ag/BiOBr_(OV)_/BiOI_0.08_ photocatalysts.

The XPS spectra of BiOBr_(OV)_/BiOI_0.08_ and 0.5% Ag/BiOBr_(OV)_/BiOI_0.08_, which elucidated the surface element types and valence states for the synthesized photocatalysts, are presented in Figure 5. Peak positions were referenced to the C 1s peak (284.8 eV), and the survey spectra of the photocatalysts indicated the detection of C, Bi, O, Br, and I in both photocatalysts and the presence of Ag in 0.5% Ag/BiOBr_(OV)_/BiOI_0.08_, confirming the high purity of the photocatalysts. The binding energies for the Ag 3d_5/2_ and Ag 3d_3/2_ spin–orbit splitting photoelectrons of 0.5% Ag/BiOBr_(OV)_/BiOI_0.08_ were 368.1 and 374.2 eV, respectively (Figure 5b), corresponding to the Ag 3d_5/2_ and Ag 3d_3/2_ binding energies of metallic Ag [49]. The 164.4 eV and 159.1 eV peaks in Figure 5c were ascribed to Bi 4f_5/2_ and Bi 4f_7/2_, respectively, and they corresponded to the characteristic binding energies of the Bi^3+^ in BiOX [10,18]. The 630.4 eV and 618.5 eV peaks in Figure 5d were attributed to the I 3d_3/2_ and I 3d_5/2_ of BiOI, respectively [10,18]. The Br 3d spectra in Figure 5e exhibit Br 3d_3/2_ (69.0 eV for 0.5% Ag/BiOBr_(OV)_/BiOI_0.08_ and 69.2 eV for BiOBr_(OV)_/BiOI_0.08_) and Br 3d_5/2_ (68.1 eV for 0.5% Ag/BiOBr_(OV)_/BiOI_0.08_ and 68.3 eV for BiOBr_(OV)_/BiOI_0.08_) peaks, indicating the presence of Br^−^ [10,18]. The O 1s spectra in Figure 5f were deconvoluted into the peak assigned to the Bi-O bonds in the [Bi_2_O_2_] slabs of the BiOX layered structure (529.8 eV for 0.5% Ag/BiOBr_(OV)_/BiOI_0.08_ and 530.1 eV for BiOBr_(OV)_/BiOI_0.08_) and the peak attributed to the O 1s species located at the OVs on the photocatalyst surface (531.1 eV for 0.5% Ag/BiOBr_(OV)_/BiOI_0.08_ and 531.5 eV for BiOBr_(OV)_/BiOI_0.08_) [18]. Overall, the surface elements (together with their valence states) discerned from the XPS spectra of BiOBr_(OV)_/BiOI_0.08_ and Ag/BiOBr_(OV)_/BiOI_0.08_ were consistent with the elemental components of the photocatalysts.

**Figure 6:**
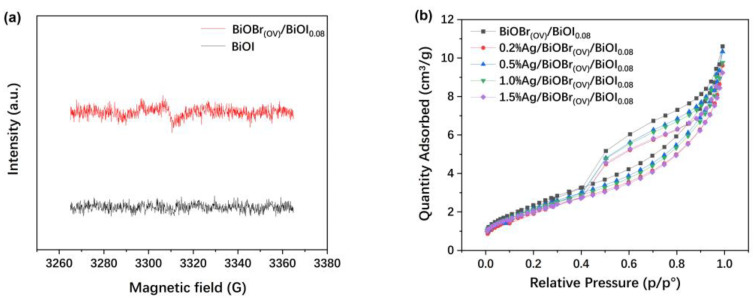
ESR spectra of BiOBr_(OV)_/BiOI_0.08_ using BiOI as a reference (**a**). N_2_ adsorption–desorption isotherms of BiOBr_(OV)_/BiOI_0.08_ and Ag/BiOBr_(OV)_/BiOI_0.08_ photocatalysts (**b**).

The ESR spectrum of BiOBr_(OV)_/BiOI_0.08_, which is depicted in Figure 6a, exhibited a prominent signal at g = 2.001, indicating that the photocatalyst contained OVs [34,35]. Conversely, this signal is absent in the BiOI sample.

According to the N_2_ adsorption–desorption isotherms shown in Figure 6b, BiOBr_(OV)_/BiOI_0.08_ had the highest specific surface area (9.36 m^2^/g), followed by 0.2% Ag/BiOBr_(OV)_/BiOI_0.08_ (8.69 m^2^/g), 0.5% Ag/BiOBr_(OV)_/BiOI_0.08_ (8.24 m^2^/g), 1.0% Ag/BiOBr_(OV)_/BiOI_0.08_ (8.01 m^2^/g), and 1.5% Ag/BiOBr_(OV)_/BiOI_0.08_ (7.82 m^2^/g), respectively, demonstrating that an increase in the Ag loading level decreased the specific surface area of Ag/BiOBr_(OV)_/BiOI_0.08_ photocatalysts.

### 3.2. Photocatalytic Activity

**Figure 7:**
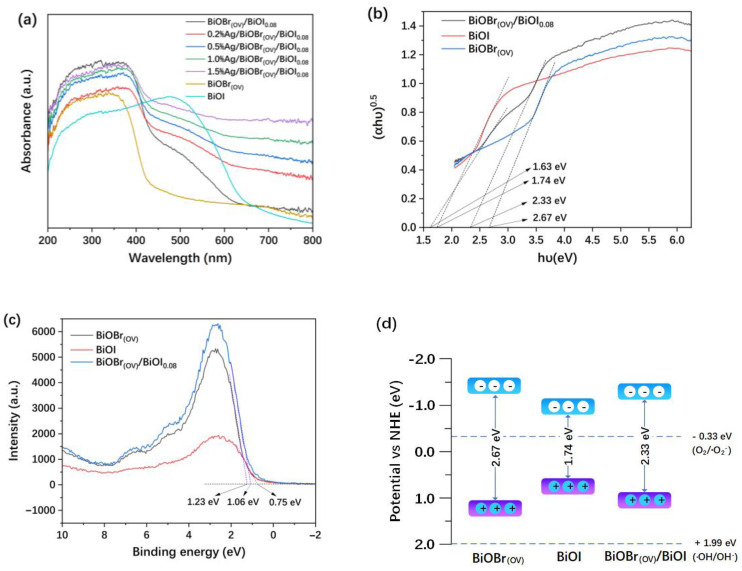
UV-vis DRS spectra (**a**). Bandgap estimation using (αhν)^0.5^ vs. h*ν* curves (**b**). VB-XPS spectra (**c**). The band position schematic (**d**).

The UV-vis DRS spectra of the synthesized photocatalysts (200–800 nm, Figure 7a) indicated that the photocatalysts had stronger ultraviolet light absorption than visible-light absorption properties. In the visible-light spectrum, the light absorption capacities of the photocatalysts markedly decreased as the wavelength increased. However, the light absorption capacity of Ag/BiOBr_(OV)_/BiOI_0.08_ photocatalysts was significantly enhanced, particularly in the visible-light spectrum, as the Ag loading level increased. Ag/BiOBr_(OV)_/BiOI_0.08_ photocatalysts darkened as the Ag loading level increased.

Bandgap values were estimated according to the Kubelka–Munk function by analyzing (αh*ν*)^0.5^ vs. photon energy (h*ν*) curves, where α, h, and ν represent the absorption coefficient, Planck’s constant, and light frequency, respectively [18,20]. As illustrated in Figure 7b, the estimated band gaps of BiOBr_(OV)_ and BiOI are 2.67 and 1.74 eV, respectively. Two different band-gap values were estimated for BiOBr_(OV)_/BiOI_0.08_ (2.33 eV and 1.63 eV). This behavior suggests that a low concentration of energy states exists within the valence and conduction bands, which can be ascribed to the presence of multiple phases of BiOI [50]. The valence band edge (E_VB_) values of BiOBr_(OV)_, BiOI, and BiOBr_(OV)_/BiOI_0.08_, which were 1.23, 0.75, and 1.06 eV, respectively, were calculated by constructing valence band X-ray photoelectron spectroscopy (VB-XPS) spectra (Figure 7c). Then, the conduction band edge (E_CB_) values of BiOBr_(OV)_, BiOI, and BiOBr_(OV)_/BiOI_0.08_ were calculated using the equation E_CB_ = E_VB_ − E_g_ [20,38], and they were −1.44, −0.99, and −1.27 eV, respectively. Based on these calculations, a slab model was constructed to visually represent the band positions and gaps of these samples, as shown in Figure 7d. Compared to BiOBr_(OV)_, BiOBr_(OV)_/BiOI_0.08_ had a narrower bandgap and a higher E_VB_, indicating its enhanced visible-light absorption capacity.

In photocatalysis, the positively charged holes (*h*^+^) are capable of oxidizing H_2_O or OH^−^ to produce ‧OH radicals. However, this oxidation can occur only if the E_VB_ of the photocatalyst is more positive than the ‧OH/OH^−^ redox potential (1.99 eV). Conversely, ‧O_2_^−^ radicals are predominantly formed through the photogenerated electrons (e^−^) captured by O_2_, which can occur only when the E_CB_ of the photocatalyst is more negative than the ‧O_2_^−^/O_2_ redox potential (−0.33 eV) [18]. The calculated E_VB_ and E_CB_ values for BiOBr_(OV)_/BiOI_0.08_ were 1.06 eV and −1.27 eV, respectively. Therefore, on the surface of this photocatalyst, the direct generation of ‧OH by *h*^+^ is very difficult, but ‧O_2_^−^ can be readily produced through electron capture. Additionally, ‧OH can be indirectly produced via ‧O_2_^−^ reduction and H_2_O_2_ decomposition. The reduction in surface-adsorbed O_2_ and subsequent oxidation of ‧O_2_^−^ are the likely mechanisms for ^1^O_2_ formation [8]. Photogenerated excitons (e^−^-*h*^+^ pairs) can be formed on the semiconductor photocatalyst surface, and their Coulomb interaction typically results in a strong exciton effect for which its associated energy transfer can convert triplet oxygen (^3^O_2_) to ^1^O_2_ [51].

**Figure 8:**
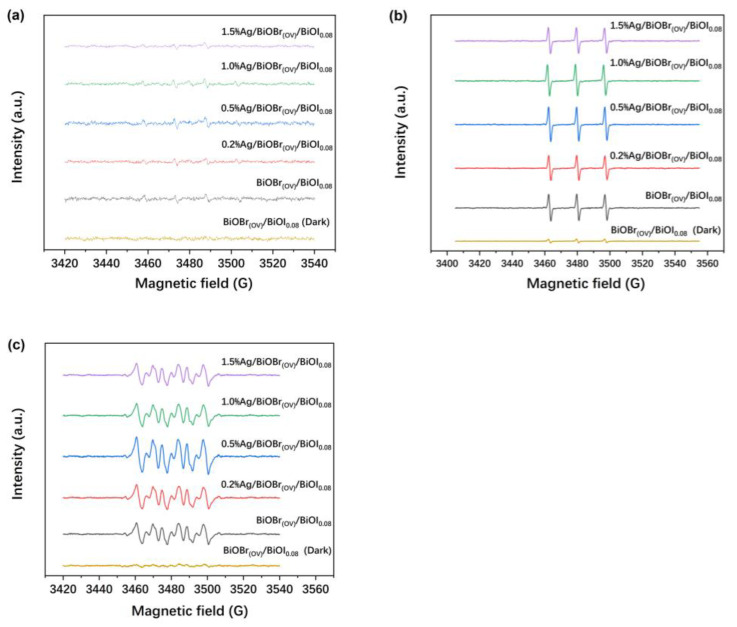
ESR spectra of DMPO-‧OH (**a**), TEMP-^1^O_2_ (**b**), and DMPO-‧O_2_^−^ (**c**) adducts (the samples irradiated by light were under visible light irradiation for 90 s, respectively).

ROS (‧OH, ^1^O_2_, and ‧O_2_^−^) generation during photodegradation was detected using ESR. The DMPO-‧OH, TEMP-^1^O_2_, and DMPO-‧O_2_^−^ ESR spectra recorded with and without light irradiation (Figure 8) indicated ‧OH, ^1^O_2_, and ‧O_2_^−^ generation in the reaction system under light irradiation. The ^1^O_2_ and ‧O_2_^−^ signals were notably strong, whereas the ‧OH signal was comparatively weak.

Without light irradiation, the ROS signals were nearly undetectable, verifying the generation of ‧OH, ^1^O_2_, and ‧O_2_^−^ during photodegradation employing BiOBr_(OV)_/BiOI_0.08_ or Ag/BiOBr_(OV)_/BiOI_0.08_. As the Ag loading level of Ag/BiOBr_(OV)_/BiOI_0.08_ photocatalysts increased, the characteristic signals of the ROS weakened.

**Figure 9:**
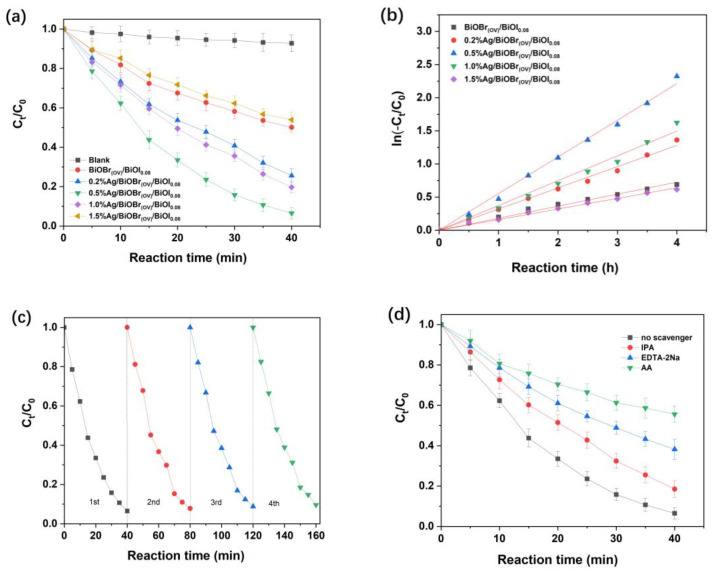
Photocatalytic performance of BiOBr_(OV)_/BiOI_0.08_ and Ag/BiOBr_(OV)_/BiOI_0.08_ photocatalysts for 4-CP degradation under visible-light irradiation (**a**). Pseudo-first-order kinetic model fitting (**b**). Cyclic stability of 0.5% Ag/BiOBr_(OV)_/BiOI_0.08_ (**c**). Effects of scavengers on 0.5% Ag/BiOBr_(OV)_/BiOI_0.08_ photocatalytic performance (**d**).

Figure 9 illustrates the efficacy of the synthesized catalysts in degrading 4-CP in aqueous solutions. Figure 9a depicts photocatalytic performance comparisons between BiOBr_(OV)_/BiOI_0.08_ and Ag/BiOBr_(OV)_/BiOI_0.08_ photocatalysts. Without a photocatalyst, 4-CP exhibited negligible degradation under visible-light irradiation. However, with BiOBr_(OV)_/BiOI_0.08_, approximately 50% of 4-CP was degraded within 4 h. The degradation efficiencies of 4-CP with catalysts loaded with 0.2%, 0.5%, 1.0%, and 1.5% Ag were 74.4%, 90.2%, 80.3%, and 46.4%, respectively. It is evident that photocatalytic efficiency does not consistently increase with higher silver loadings, with the 0.5% Ag/BiOBr_(OV)_/BiOI_0.08_ catalyst demonstrating the highest photocatalytic performance.

Pseudo-first-order kinetics were illustrated in −ln(*C_t_*/*C*_0_) vs. irradiation time (*t*) plots (Figure 9b). The apparent rate constant (*k*) for 4-CP degradation using 0.5% Ag/BiOBr_(OV)_/BiOI_0.08_ was 0.55 h⁻^1^, which was 3.0, 1.7, 1.5, and 3.4 times greater than those of BiOBr_(OV)_/BiOI_0.08_ (0.18 h^−1^), 0.2% Ag/BiOBr_(OV)_/BiOI_0.08_ (0.32 h⁻^1^), 1.0% Ag/BiOBr_(OV)_/BiOI_0.08_ (0.37 h⁻^1^), and 1.5% Ag/BiOBr_(OV)_/BiOI_0.08_ (0.16 h⁻^1^), respectively. The recyclability of the 0.5% Ag/BiOBr_(OV)_/BiOI_0.08_ nanoparticles for 4-CP degradation is depicted in Figure 9c, indicating that the catalyst maintained relatively stable photocatalytic activity after four cycles of usage.

The 4-CP removal mechanism of 0.5% Ag/BiOBr_(OV)_/BiOI_0.08_ was elucidated by employing isopropanol (IPA), ethylenediaminetetraacetic acid disodium salt (EDTA-2Na), and ascorbic acid (AA) as ‧OH, ‧O_2_^−^, and ^1^O_2_ trapping agents, respectively [18,52]. After IPA (2 mM), EDTA-2Na, or AA incorporation, the 4-CP removal efficiency of 0.5% Ag/BiOBr_(OV)_/BiOI_0.08_ decreased from 90.2% to 81.5%, 61.8%, or 44.4%, respectively, suggesting that ‧O_2_^−^ and ^1^O_2_, instead of ‧OH, were the critical active species in 0.5% Ag/BiOBr_(OV)_/BiOI_0.08_ photocatalytic activity, with *h*⁺ playing an essential role in 4-CP photocatalytic removal under visible-light irradiation.

### 3.3. PCC Separation

**Figure 10:**
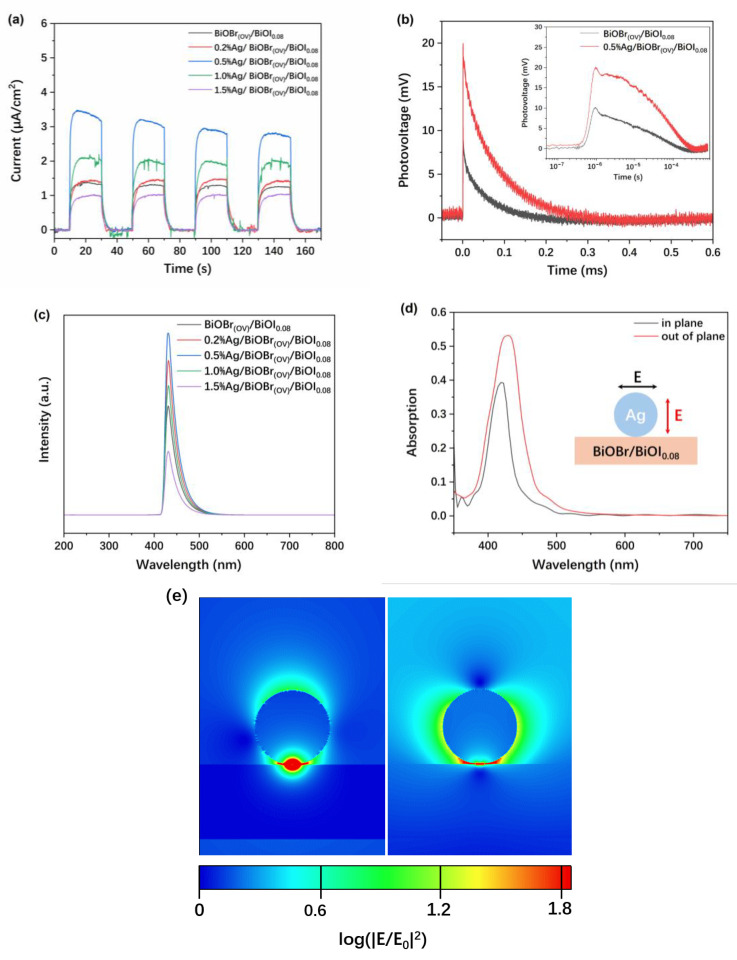
Surface photocurrent (SPC) of different samples (**a**). TPV spectra of BiOBr_(OV)_/BiOI_0.08_ and 0.5% Ag/BiOBr_(OV)_/BiOI_0.08_ recorded under 355 nm pulsed-laser irradiation (**b**). PL spectra of BiOBr_(OV)_/BiOI_0.08_ and Ag/BiOBr_(OV)_/BiOI_0.08_ photocatalysts (**c**). Simulated absorption spectra (**d**) and EF intensity enhancement contours (**e**) of a Ag/BiOBr/BiOI_0.08_ system consisting of a Ag nanosphere (with a 30 nm diameter) supported on a BiOBr/BiOI_0.08_ nanosheet (with a 15 nm thickness). The inset in (**d**) depicts the in-plane (black) and out-of-plane (red) polarized excitations (relative to the support). The left and right images in (**e**) were recorded under the out-of-plane and in-plane polarized excitations, respectively.

Surface photocurrent (SPC) intensity and PCC separation are correlated. Therefore, the photocurrent responses of BiOBr_(OV)_/BiOI_0.08_ and Ag/BiOBr/BiOI_0.08_ photocatalysts under visible-light (λ > 400 nm) irradiation were measured. Figure 10a illustrates the SPC response of the different samples. The photocurrent was nearly zero when the light was off, while it was distinct and stable when the light was on, signifying that all synthesized samples possessed PCC separation capability. Under identical illumination conditions, varying samples demonstrated different photocurrent intensities. Generally, a higher photocurrent intensity indicates superior photogenerated charge separation efficiency. In comparison to BiOBr_(OV)_/BiOI_0.08_, catalysts with 0.2%, 0.5%, and 1.0% Ag loading exhibited enhanced photocurrent responses. This enhancement suggests that the loading of a small quantity of Ag nanoparticles facilitates PCC separation and thereby augments photocurrent intensity. However, with a further increase in silver nanoparticle loading, a decline in photocurrent was observed. Notably, compared to BiOBr_(OV)_/BiOI_0.08_ and 1.0% Ag/BiOBr_(OV)_/BiOI_0.08_, 1.5% Ag/BiOBr_(OV)_/BiOI_0.08_ exhibited significantly lower photocurrent responses, indicating that an excessive Ag loading level is detrimental to PCC separation. The plausible explanation is that an excessive amount of silver may cover the effective surface area of the semiconductor, thereby impeding light absorption. Another critical reason could be that excessive silver loading alters the surface band structure of the catalyst, fostering PCC recombination and thus diminishing SPC. These results suggest that a minimal loading (not exceeding 1%) of silver nanoparticles significantly enhances the catalytic activity of the BiOBr_(OV)_/BiOI_0.08_ catalyst.

TPV spectra can provide information on PCC generation, separation, and recombination. The TPV spectra of BiOBr_(OV)_/BiOI_0.08_ and 0.5% Ag/BiOBr_(OV)_/BiOI_0.08_ were measured to investigate the dynamics of PCC transfer processes. Figure 10b shows the TPV spectra of BiOBr_(OV)_/BiOI_0.08_ and 0.5% Ag/BiOBr_(OV)_/BiOI_0.08_ excited by 355 nm light. From the TPV spectra of 0.5% Ag/BiOBr_(OV)_/BiOI_0.08_, there are two photogenerated charge transfer processes after the sample is excited by light. The first one was a quick process spanning the time range of 10^−7^ to 10^−6^ s, representing PCC separation in BiOBr/BiOI_0.08_ crystallites. The second one was a slower process spanning the time range of 10^−6^ to 10^−5^ s, representing PCC transfers between particles and dependent on the photocatalyst composition and structure. The PCC transfer time of 0.5% Ag/BiOBr_(OV)_/BiOI_0.08_ was significantly prolonged compared to that of BiOBr_(OV)_/BiOI_0.08_, and it was attributed to the required time for PCC transfer between BiOBr_(OV)_/BiOI_0.08_ crystallites and Ag nanoparticles. This observation indicates that an appropriate Ag loading level facilitates an efficient photocatalytic process because it not only enhances PCC separation but also extends the PCC lifetime.

The PL emission intensities of Ag/BiOBr_(OV)_/BiOI_0.08_ photocatalysts with Ag loading levels not more than 1% were significantly higher than that of BiOBr_(OV)_/BiOI_0.08_. Especially for the 0.5% Ag loading, the PL emission intensity reached about 1.7 times the original level (Figure 10c). However, 1.5% Ag loading significantly decreased the PL emission intensity. A possible reason for this is that excessive Ag nanoparticles cover the Ag/BiOBr_(OV)_/BiOI_0.08_ surface, reducing light absorption and PCC generation in the photocatalyst.

The EF intensity distribution on Ag/BiOBr_(OV)_/BiOI_0.08_ nanoparticles was investigated via FDTD simulations to ascertain whether the enhanced PL emission intensity of the composite nanoparticles was due to local EF enhancements in the near-field regions surrounding Ag nanoparticles.

The refractive indices of BiOBr and BiOI were sourced from the relevant literature [46]. The LSPR peaks for the in-plane and out-of-plane polarized excitations were detected in the simulated absorption spectra of the Ag/BiOBr/BiOI_0.08_ system at 420 nm and 430 nm, respectively (Figure 10d). The absorption curve derived from the simulation closely aligns with the experimental results.

LSPR significantly enhanced the EF intensity at the interface between the Ag nanosphere and BiOBr/BiOI_0.08_ nanosheet (Figure 10e), concentrating incident light energy and forming high-energy-density “hot spots” where trapped *e*^−^ were readily excited to the CB of BiOBr/BiOI_0.08_ [53]. The square of the EF intensity enhancement was calculated to characterize the near-field enhancement effect of local plasmons, and it reached 65 at the “hot spots”.

### 3.4. DFT Calculation

**Figure 11:**
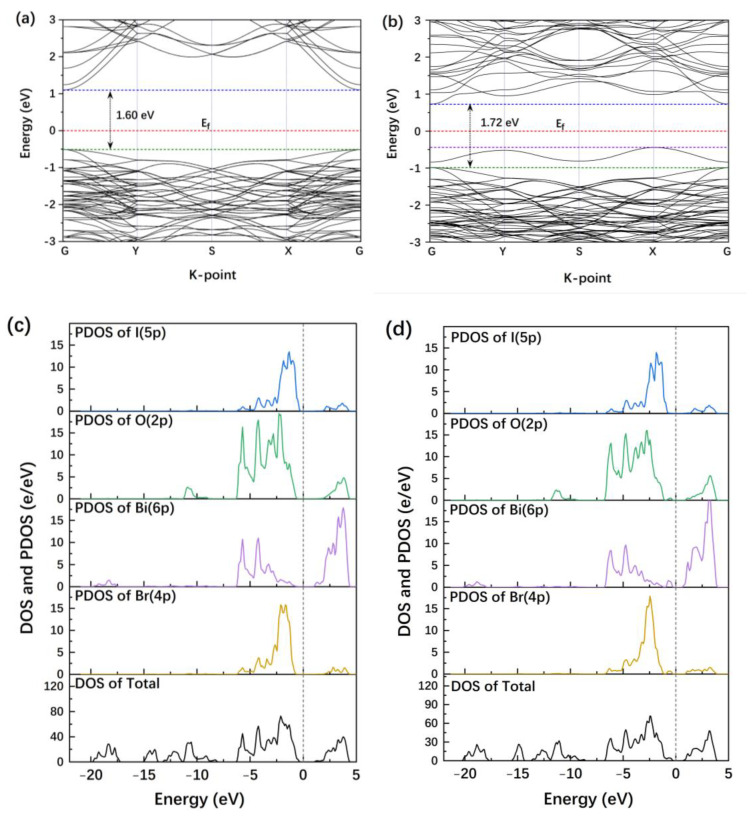

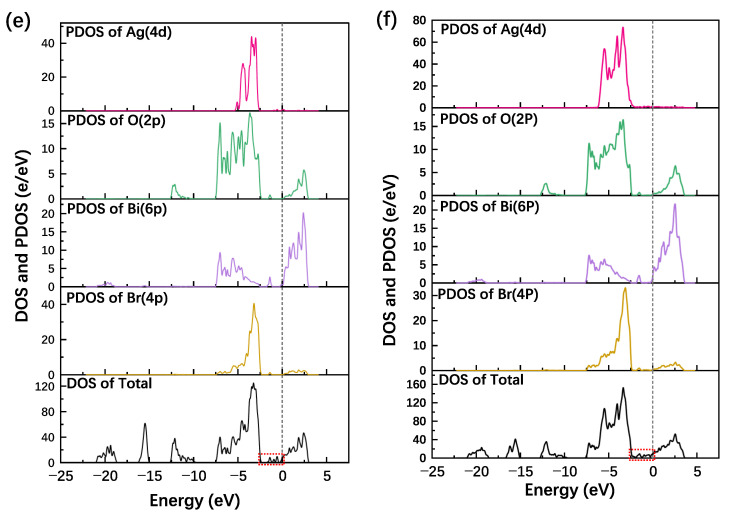
Band structures of BiOBr/BiOI (**a**) and BiOBr_(OV)_/BiOI (**b**). TDOS and PDOS spectra of BiOBr/BiOI (**c**), BiOBr_(OV)_/BiOI (**d**), Ag(cluster)/BiOBr_(OV)_ (**e**), and Ag(layer)/BiOBr_(OV)_ (**f**).

The influence of OVs on the band structure of BiOBr/BiOI heterojunctions was assessed by conducting DFT calculations to obtain the band structure, total density of states (TDOSs), projected density of states (PDOSs), and high symmetry points in the Brillouin zone for each of BiOBr/BiOI and BiOBr_(OV)_/BiOI (Figure 11).

The Fermi levels for BiOBr/BiOI and BiOBr_(OV)_/BiOI (Figure 11a,b) were located at 0 eV, and the indirect bandgaps of the photocatalysts were 1.60 eV and 1.72 eV, respectively. The CB and VB of each photocatalyst were located above and below the Fermi level, respectively. As BiOBr_(OV)_/BiOI contained OVs, its VB and CB were shifted to lower energy levels compared to those of BiOBr/BiOI. Moreover, the existence of OVs increased the bandgap of BiOBr/BiOI heterojunctions from 1.60 eV (for BiOBr/BiOI) to 1.72 eV (for BiOBr_(OV)_/BiOI) and concurrently disrupted the periodic potential field formed by periodically arranged atoms, creating an impurity energy level between the VB and CB of BiOBr_(OV)_/BiOI. This impurity energy level was located below the Fermi level and near the top of the VB, and it was advantageous for PCC separation and BiOBr_(OV)_/BiOI photocatalytic performance. The VB of BiOBr_(OV)_/BiOI was composed of O 2p, Br 4p, and I 5p orbitals hybridized with Bi 6p orbitals, while the CB of the photocatalyst was contributed by Bi 6p orbitals that were slightly hybridized with O 2p, Br 4p, and I 5p orbitals (Figure 11d). The impurity energy level was composed of Bi 6p orbitals hybridized with adjacent Br 4p and O 2p orbitals.

In order to elucidate the effect of Ag loading on the BiOBr_(OV)_, the DFT was also carried out to calculate the DOS of the semiconductor surface loaded with Ag atomic clusters composed of six silver atoms (Figure S3). In Ag_(cluster)_/BiOBr_(OV)_, the Ag 4d and Bi 6p states intersect the Fermi level. The VB primarily consisted of Ag 4d, O 2p, and Br 4p orbitals, with minor contributions from Bi 6p orbitals, while the CB was mainly contributed by Bi 6p orbitals, with slight contributions from O 2p and Br 4p orbitals (Figure 11e).

The presence of OV and the silver cluster results in the formation of multiple impurity levels within the bandgap, located below the Fermi level. Some impurity levels are proximate to the bottom of the CB, potentially stabilizing holes generated by Ag nanoparticles via plasmon resonance [54]. Compared to BiOBr_(OV)_, the bandgap width of Ag_(cluster)_/BiOBr_(OV)_ is significantly reduced, resulting in a redshift of the absorption band edge.

Increasing silver loading may lead to larger silver particle sizes due to agglomeration. It can be hypothesized that within a limited range, the BiOBr_(OV)_ surface is covered by a silver atomic layer. Consequently, the band structure of BiOBr_(OV)_ loaded with a silver atomic layer was also calculated using DFT (Figure S4). The VB and CB energy levels of Ag_(layer)_/BiOBr_(OV)_ overlapped, and the bandgap disappeared (Figure 11f and Figure S5). This implies that the semiconductor loaded with a Ag atomic layer exhibits metallicity, which might facilitate the rapid recombination of PCC, but it is detrimental to its photocatalytic activity. Therefore, compared to a high Ag loading level, a low Ag loading level was more effective in promoting PCC separation and thereby improving Ag/BiOBr_(OV)_/BiOI_0.08_ photocatalytic performance.

### 3.5. The Ag/BiOBr_(OV)_/BiOI_0.08_ Photocatalytic Mechanism

**Figure 12:**
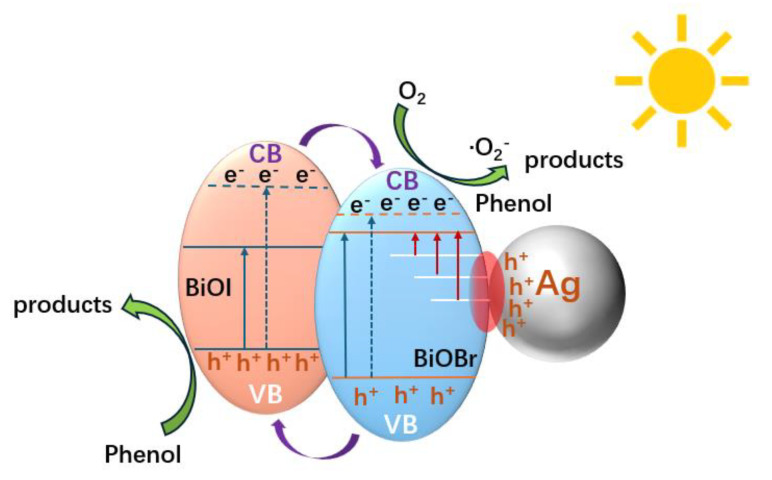
The PCC transfer process of Ag/BiOBr_(OV)_/BiOI_0.08_ photocatalysts under visible-light irradiation.

The CB and VB potentials of BiOI and BiOBr_(OV)_ suggest that the BiOBr_(OV)_/BiOI heterojunction is a type I heterojunction (Figure 12). The presence of BiOI enhances the visible light absorption of the composite catalyst. The *e*^−^ on the BiOI VB can be excited to the BiOI CB under visible radiation and then transferred to the BiOBr_(OV)_ CB. They could react with adsorbed O_2_ on the BiOBr_(OV)_/BiOI surface, producing ‧O_2_^−^ and H_2_O_2_, which could degrade organic compounds. The *h*^+^ on the BiOBr_(OV)_ VB could migrate to the BiOI VB and oxidize organic pollutants. The LSPR of Ag nanoparticles also significantly enhances visible light absorption via the catalyst. Impurity energy levels generated by the introduction of OVs and loading of Ag nanoparticles could trap plasmonic hot electrons, and the plasmon-induced “hot spots” formed at the interface between Ag nanoparticles and the BiOBr_(OV)_/BiOI semiconductor greatly enhanced the EF intensity in the near-field region, promoting *e*^−^ excitation from the “hot spots” to the BiOBr_(OV)_ CB. The plasmonic *h*^+^ remaining in Ag nanoparticles could also contribute to pollutant oxidation.

## 4. Conclusions

In this work, Ag/BiOBr_(OV)_/BiOI_0.08_ photocatalysts were successfully constructed using a straightforward hydrothermal and photo-reduction route. About 0.5% of Ag loading can significantly improve the catalytic ability of BiOBr_(OV)_/BiOI_0.08_. The 0.5% Ag/BiOBr_(OV)_/BiOI_0.08_ photocatalyst demonstrated an exceptional visible-light-driven 4-CP degradation performance, with ‧O_2_^−^, ^1^O_2_, and *h*^+^ being important active species. FDTD simulation results showed the LSPR-induced formation of “hot spots” at the interface between Ag nanospheres and the BiOBr_(OV)_/BiOI_0.08_ semiconductor. At the “hot spots”, trapped electrons were readily excited to the CB of BiOBr_(OV)_. DFT calculation results confirmed that OVs and a low Ag loading level facilitated the formation of multiple impurity energy levels in the bandgap of Ag/BiOBr_(OV)_/BiOI_0.08_ photocatalysts, promoting the trapping and transfer of plasmonic hot electrons. However, an increase in the size of silver particles imparts metallic characteristics to the catalyst, which detrimentally affects its photocatalytic performance. This study not only demonstrates the promising application of Ag/BiOBr_(OV)_/BiOI_0.08_ photocatalysts in visible-light-driven pollutant photocatalytic degradation but also provides guidance for synthesizing high-performance visible-light-sensitive photocatalysts.

## Data Availability

The original contributions presented in this study are included in the article/Supplementary Material. Further inquiries can be directed to the corresponding author.

## Supplementary Materials

The following supporting information can be downloaded at https://www.mdpi.com/article/10.3390/ma17246297/s1. Figure S1: The structure of BiOBr/BiOI used for simulation calculation; Figure S2: The structure of BiOBr_(OV)_/BiOI used for simulation calculation; Figure S3: The structure of Ag(cluster)/BiOBr_(OV)_ used for simulation calculation; Figure S4: The structure of Ag(layer)/BiOBr_(OV)_ used for simulation calculatio; Figure S5: The band structure and projected density of states (PDOS) of Ag(layer)/BiOBr_(OV)_.
