# Supplementary material for: New Insight into Visible-Light-Driven Photocatalytic Activity of Ag-Loaded and Oxygen Vacancy-Containing BiOBr(OV)/BiOI0.08 Microspheres
Source: Materials (Basel). 2024 Dec 23;17(24):6297. doi: 10.3390/ma17246297 (PMC11678820; doi:10.3390/ma17246297)
Supplement: Supplementary file 1 [file materials-17-06297-s001.zip › materials-3301589-supplementary.pdf]

Supplementary Material

New Insight into Visible-Light-Driven Photocatalytic Activity of Ag-Loaded and Oxygen Vacancy-Containing BiOBr(OV)/BiOI0.08 Microspheres

Xiaobin Hu *, Mingxing Zhao, Rongfei Zhang

*Email: xiaobinhu001@163.com

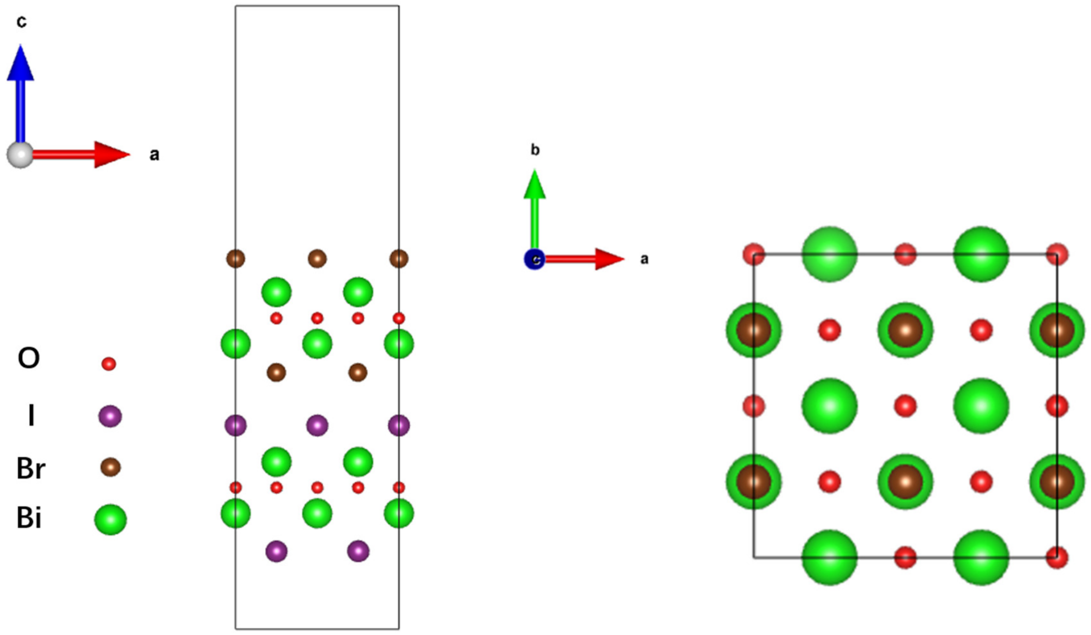

Figure S1. The structure of BiOBr/BiOI used for simulation calculation.

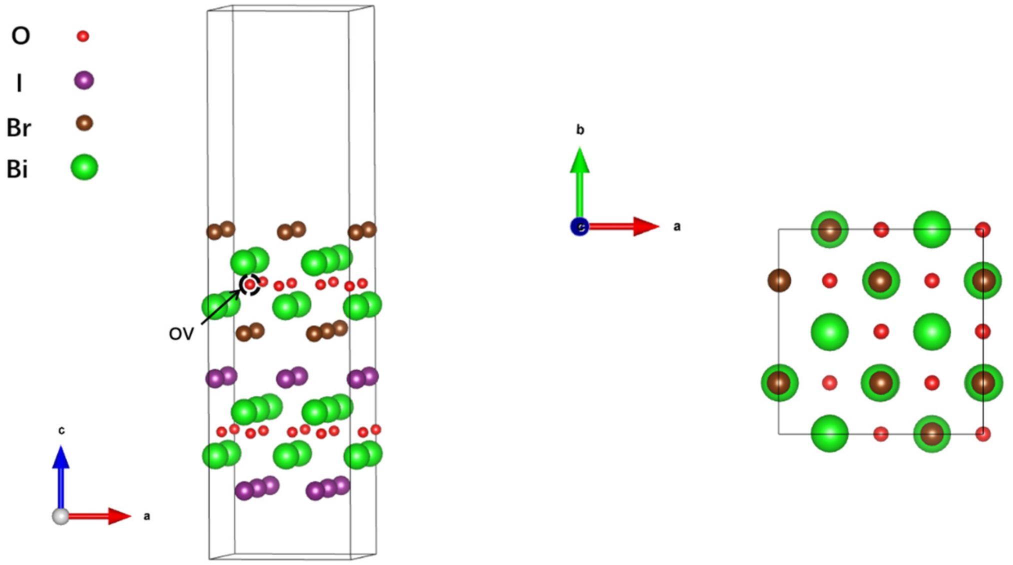

Figure S2. The structure of $BiOBr_{(OV)}$/BiOI used for simulation calculation.

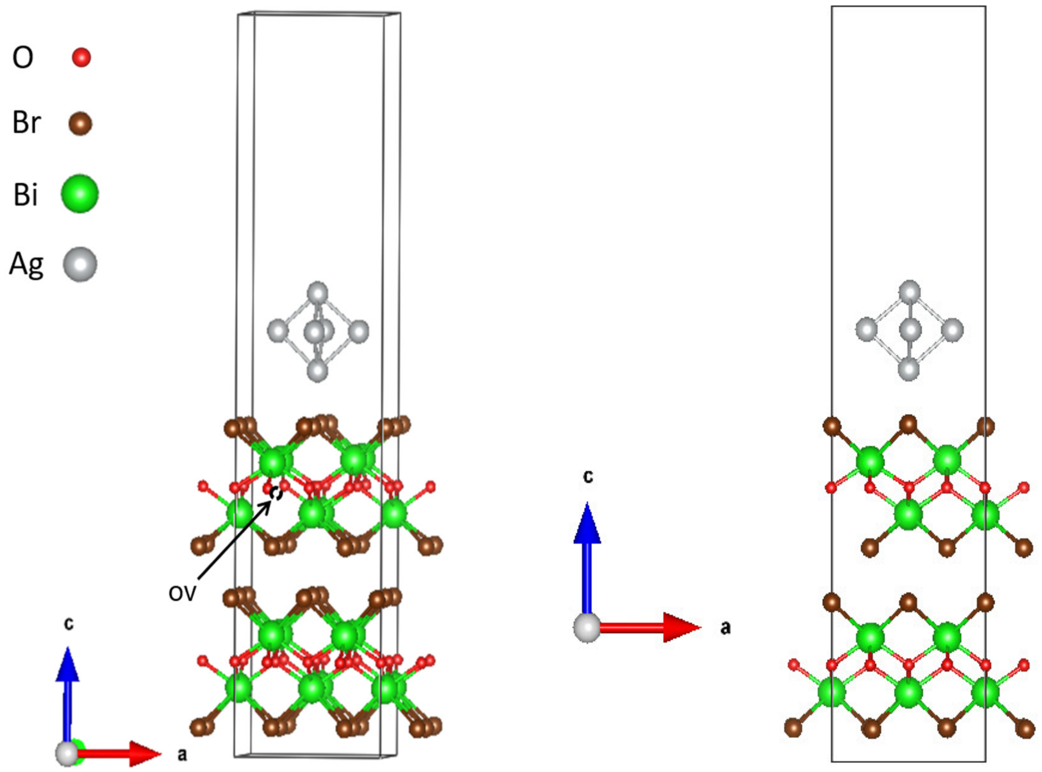

Figure S3. The structure of Ag(cluster)/$BiOBr_{(OV)}$ used for simulation calculation.

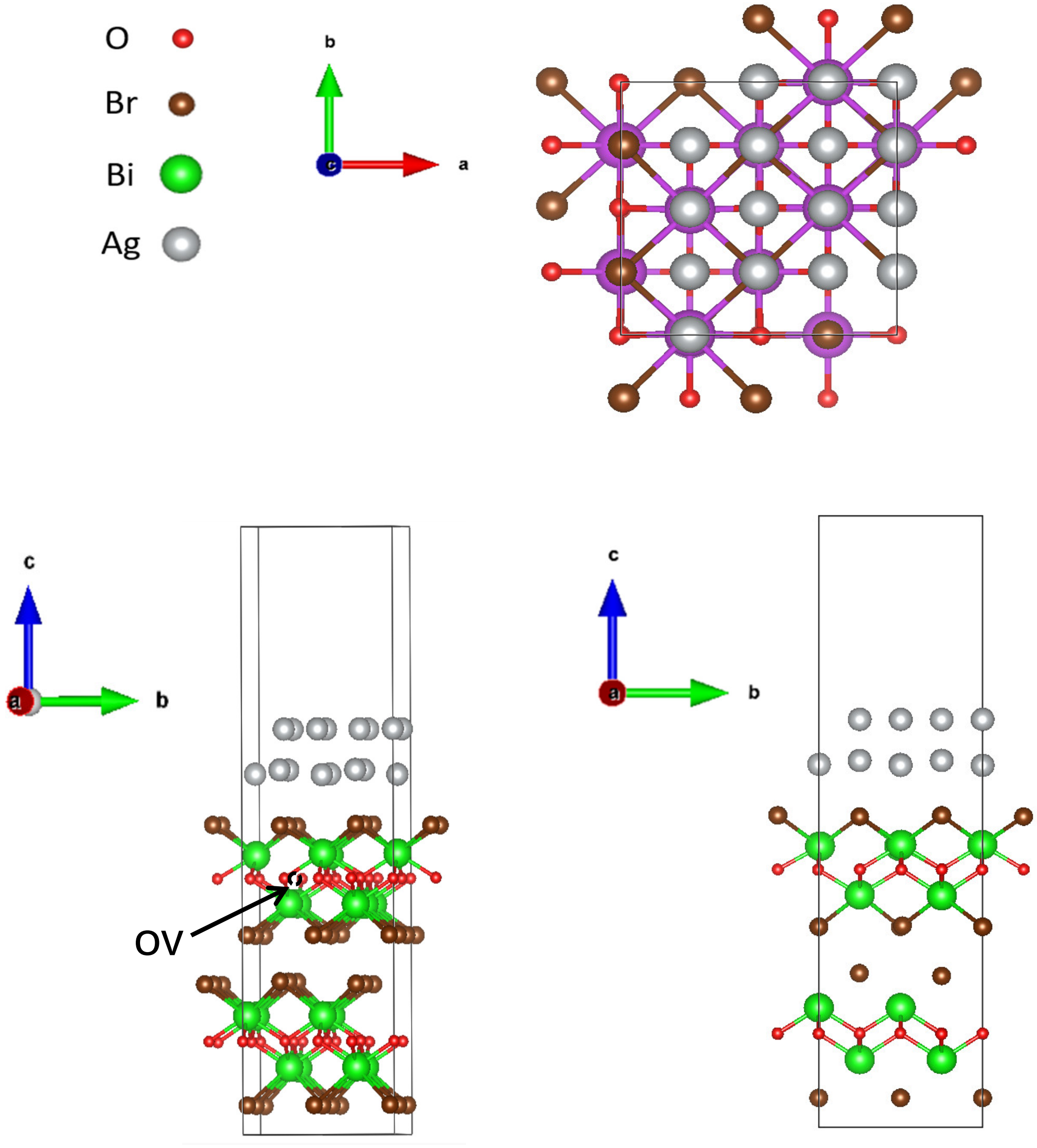

Figure S4. The structure of Ag(layer)/$BiOBr_{(OV)}$ used for simulation calculation.

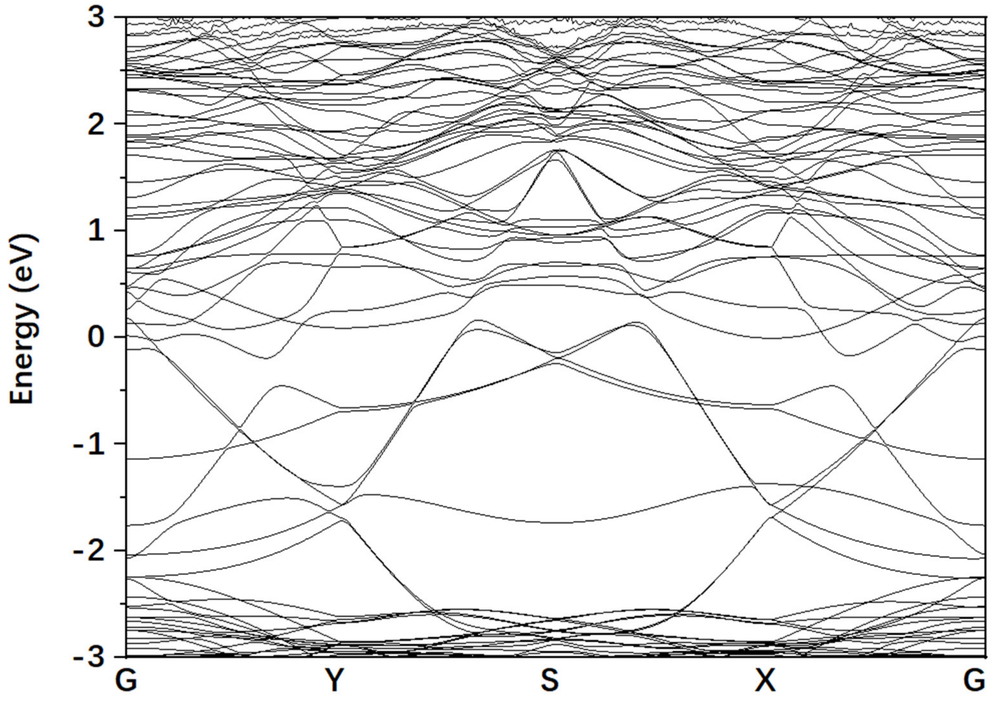

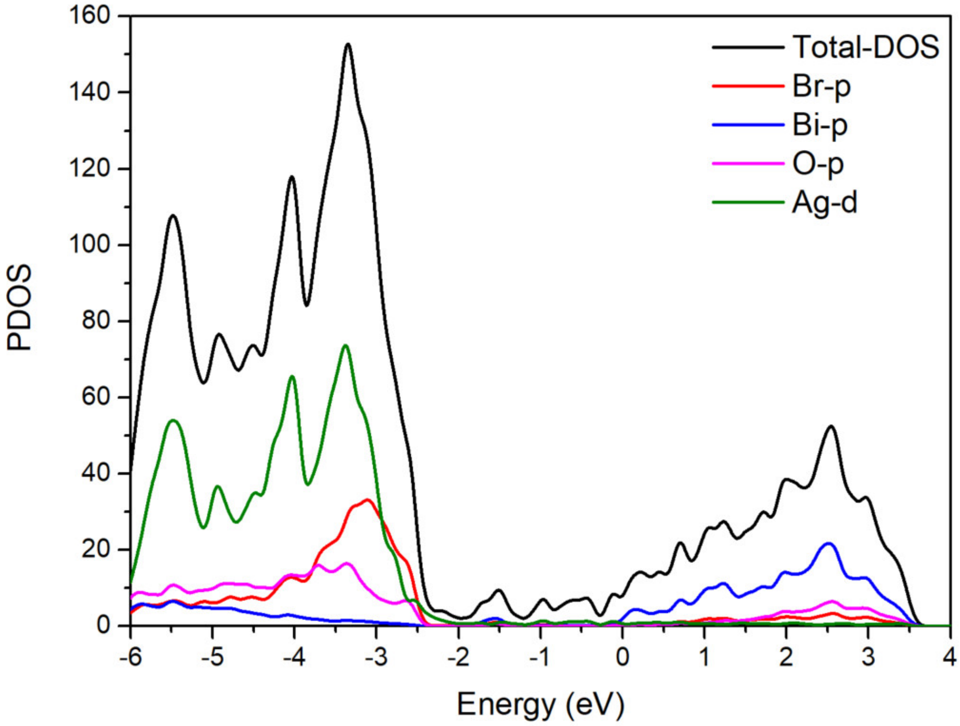

Figure S5. The band structure and projected density of states (PDOS) of Ag(layer)/$BiOBr_{(OV)}$.
